# Gender and grade differences in objectively measured physical activity and sedentary behavior patterns among Japanese children and adolescents: a cross-sectional study

**DOI:** 10.1186/s12889-015-2607-3

**Published:** 2015-12-18

**Authors:** Kaori Ishii, Ai Shibata, Minoru Adachi, Keiko Nonoue, Koichiro Oka

**Affiliations:** Faculty of Sport Sciences, Waseda University, 2-579-15 Mikajima, Tokorozawa, Saitama 359-1192 Japan; Faculty of Health and Sport Sciences, University of Tsukuba, 1-1-1 Tennodai, Tsukuba, Ibaraki 305-8575 Japan; Graduate School of Education, Okayama University, 1-1-1 Tsushima-naka, Kita, Okayama 700-8530 Japan; Sonan Junior High School, 130-2 Fujisaki, Naka, Okayama 702-8006 Japan

**Keywords:** Exercise, Children, Accelerometry

## Abstract

**Background:**

Physical activity levels in childhood have decreased, making the promotion of children’s physical activity an important issue. The present study examined gender and grade differences in objectively measured sedentary behavior, physical activity, and physical activity guideline attainment among Japanese children and adolescents.

**Methods:**

In total, 329 boys and 362 girls age 3–15 years completed the survey. School grade, gender, height, and weight were collected by questionnaires and physical activity objectively measured using an accelerometer (Lifecorder Suzuken Co.). Physical activity level (in MET) was classified as sedentary (<1.5), light (≥1.5 to <3), moderate (≥3 to <6), or vigorous (≥6). Continuous zero accelerometer counts for ≥20 min were censored and a valid accelerometry study required at least 3 days (2 weekdays and 1 weekend day) with > 600 min/day total wear time. Two-way analysis of covariance and logistic regression analyses, adjusted for weight status and accelerometer wear time, were used to examine gender and grade differences in physical activity variables and the likelihood of physical activity guideline attainment by gender and grade level.

**Results:**

Participants were sedentary 441.4 (SD, 140.1) min/day or 53.7 % of the average daily accelerometer wear time of 811.2 (118.7) min, engaged in light physical activity 307.1 (70.0) min or 38.4 % of wear time, moderate physical activity 34.6 (14.8) min (4.3 %), vigorous physical activity 28.3 (19.1) min (3.6 %), and took 12462.6 (4452.5) steps/day. Boys were more physically active and took more steps/day than girls. Students in higher grades were less active than those in lower grades. Boys were significantly more likely to meet physical activity guidelines than girls (OR: 2.07, 95 % CI: 1.45–2.96). Preschoolers (6.66, 4.01–11.06), lower-grade elementary school students (17.11, 8.80–33.27), and higher-grade elementary school students (7.49, 4.71–11.92) were more likely to meet guidelines than junior high school students.

**Conclusions:**

Boys and lower-grade students engaged in more physical activity and were more likely to attain guidelines than girls and higher-grade students. These findings highlight the need for effective and sustainable strategies to promote physical activity in Japanese school children.

## Background

Physical activity is important for good physical and mental health among children [1–3]. However, a recent review of longitudinal studies from around the world concluded that the level of physical activity in childhood has decreased, with a corresponding increase in adiposity [4]. The World Health Organization recommends that children and adolescents aged 6–17 years engage in at least 60 min of moderate to vigorous physical activity daily [5]. Another study recommends that boys take an average of 13,000–15,000 steps/day and girls 11,000–12,000 steps/day [6].

Before an effective intervention strategy for promoting physical activity can be developed, an understanding of current levels of physical activity (frequency, duration, intensity) in the target population is needed [7]. This assessment would allow us to identify which groups of children should be targeted for promotion of physical activity. Descriptive epidemiological studies assessing objectively measured physical activity among children suggest that boys are more active than girls and that physical activity declines in both genders with age, while sedentary behavior is higher in girls and increases in both genders with age [8–11]. These differences have been reported consistently in multiple countries [12], even though physical activity habits differ by culture and lifestyle [13].

Only a few such studies in Japanese children have been reported, and most have used self-reports to assess gender and age differences in physical activity. However, recent evidence indicates that self-reports may underestimate activity. Furthermore, self-reports do not provide an adequate description of the duration and intensity of physical activity [14, 15], although they are useful for assessing where and what type of physical activity the children are engaged in [16]. Recently, devices such as pedometers and accelerometers have been introduced to objectively measure and detect children’s physical activity. These devices provide objective measurements, and so are of much greater utility for assessing the duration and intensity of physical activity.

Identifying the characteristics of objective physical activity may help in designing more effective intervention strategies for children and adolescents. To our knowledge, no study has used such devices to objectively measure gender and age differences in daily physical activity among children and adolescents in Japan. Therefore, it is meaningful for promoting physical activity in Japanese children and adolescents. The purpose of the present study was to examine gender and grade differences in objectively measured physical activity, sedentary behavior, and attainment of physical activity guidelines among Japanese children and adolescents. The present study hypothesized that physical activity level would be lower on weekends, especially among girls and children in upper grades, and conversely that sedentary behavior would be higher on weekends.

## Methods

### Participants and data collection

The present cross-sectional study was conducted in a cohort of children attending a public preschool (3 grades, age 3–5 years), an elementary school (6 grades, age 6–11 years), and a junior high school (3 grades, age 12–14 years) in Okayama city, Japan, in 2010 and 2011. In total 2815 children (1474 boys and 1341 girls) age 3–15 years completed the survey. A total of 787 agreed to participate in the physical activity (accelerometry) component of the study, but 96 had missing physical activity data. Thus, 691 children and adolescents (329 boys and 362 girls) with valid physical activity data were included in the final analyses. The study area was neither metropolitan nor rural, with a population of 24,973 (11,986 male) in 9794 households as of June 2010 [17]. After permission to conduct the study was received from the principals of each participating school through the city Board of Education, children and their parents/guardians were sent a letter explaining the ethical considerations of the study and requesting their participation. Return of the letter constituted informed consent. Children and adolescents received the accelerometer from their classroom teachers. The study was approved by the Human Research Ethics Committee of Waseda University (application number 2011-055, 2011-255).

### Measures

#### Demographic data

Data on gender, age, height, and weight were gathered from each school. Body mass index was calculated from the height and weight data. Definitions of healthy weight, overweight, and obesity are based on Cole et al. [18].

#### Physical activity and sedentary behavior

Physical activity and sedentary behavior were measured for 7 consecutive days using a waist-worn accelerometer (Lifecorder, Suzuken Co., Ltd., Japan) widely used by individuals, from children to the elderly, in Japan. A previous study among Japanese children reported that physical activity energy expenditure using the doubly labeled water method was significantly correlated with physical activity intensity as measured by the Lifecorder (sedentary; r = −0.78, light to moderate physical activity; r = 0.71, vigorous physical activity; r = 0.83) [19]. The epoch length of Lifecorder is 2 min. Children were instructed to wear the accelerometer throughout the day except during sleep and water-related activities (e.g., bathing, swimming). The mean time of physical activity during one day was evaluated. On the basis of the nine exercise intensity levels from the Lifecorder data, physical activity was classified as sedentary (0–0.5; <1.5 MET), light (1–3; ≥1.5 to <3 MET), moderate (4–6; ≥3 to <6 MET), or vigorous (7–9; ≥6 MET) [20]. The average number of steps, time spent in sedentary behavior, and times spent in physical activities at each of the defined intensity levels were determined. Times spent in sedentary behavior and in the defined levels of physical activity are expressed as a percentage of average total daily accelerometer wear time. Continuous zero counts of ≥20 min were excluded as non-wear periods. Participants were included for analysis if they had complete data on a minimum of 3 days (2 weekdays and 1 day on the weekend) and wear time was more than 600 min on each of the 3 days. Lifecorder data were edited and aggregated using the Data Conversion Software (Knack Technical System, Inc.).

### Statistical analyses

Two-way analysis of covariance was used to examine gender and grade differences in physical activity and sedentary behavior variables separately for weekdays and weekends, adjusted for weight status and wear time. Effect sizes (η2) were also calculated to examine the practical significance of these differences. Logistic regression analyses were conducted to examine independent relationships between attainment of physical activity guidelines (recommending that children and adolescents engage in at least 60 min of moderate to vigorous physical activity daily) and both gender and grade level, adjusted for weight status and wear time. These physical activity guidelines are applied for children 6–17 years by the World Health Organization [5] and for early childhood in Japan [21]. All statistical analyses were performed using PAWS Statistics 21 and results were considered significant at *p* < 0.05.

## Results

### Demographic and physical activity of the participants

Table 1 shows the sociodemographic characteristics and physical activity levels of the study participants. The majority of participants were in junior high school (52.0 %) with the rest divided between preschool (15.2 %), lower grades of elementary school (12.7 %), and higher grades of elementary school (19.7 %). About 90 % of the participants were defined as healthy weight (BMI < 25 kg/m^2^). Time spent (mean [SD] min per day) in sedentary behavior accounted for 53.7 % (441.4 [140.1] min) of the average daily wearing time (811.2 [118.7] min) for the entire cohort. Light physical activity accounted for 38.4 % of the wearing time (307.1 [70.0] min), while only 4.3 % of the time was spent on moderate physical activity (34.6 [14.8] min) and 3.6 % on vigorous physical activity (28.3 [19.1] min). The average number of steps per day was 12462.6 [4452.5].

**Table 1:** Demographic distribution and accelerometry of the study population

		Overall		Boy		Girl	
		n	%	n	%	n	%
		691	100.0	329	47.6	362	52.4
Grade level							
	Preschool	105	15.2	51	15.5	54	14.9
	Lower grades of elementary school	88	12.7	36	10.9	52	14.4
	Higher grades of elementary school	136	19.7	68	20.7	68	18.8
	Junior high school	362	52.0	174	52.9	188	51.9
Weight status							
	Healthy weight						
	Preschool	100	95.2	50	98.0	50	92.6
	Lower grades of elementary school	80	90.9	34	94.4	46	88.5
	Higher grades of elementary school	115	84.5	55	80.9	60	88.2
	Junior high school	329	90.9	158	90.8	171	91.0
	Overweight or obese						
	Preschool	5	4.8	1	2.0	4	7.4
	Lower grades of elementary school	8	9.1	2	5.6	6	11.5
	Higher grades of elementary school	21	15.4	13	19.1	8	11.8
	Junior high school	33	9.1	16	9.2	17	9.0
Sedentary behavior, min/day							
	Mean ± SD	441.4 ± 140.1		408.6 ± 122.2		471.3 ± 148.5	
Light intensity physical activity, min/day							
	Mean ± SD	307.1 ± 70.0		315.3 ± 67.4		299.7 ± 71.6	
Moderate physical activity, min/day							
	Mean ± SD	34.6 ± 14.8		35.8 ± 15.3		33.5 ± 14.2	
Vigorous physical activity, min/day							
	Mean ± SD	28.3 ± 19.1		31.4 ± 22.0		25.4 ± 15.4	
Steps/day							
	Mean ± SD	12462.6 ± 4452.5		14010.6 ± 4628.2		11048.7 ± 3771.4	
Wearing time/day							
	Mean ± SD	811.2 ± 118.7		790.9 ± 107.7		829.7 ± 125.3	
Expressed as a percentage of the wear time, %							
	Sedentary activity	53.7		51.2		56.0	
	Light intensity physical activity	38.4		40.2		36.7	
	Moderate physical activity	4.3		4.6		4.1	
	Vigorous physical activity	3.6		4.0		3.1	

*SD* standard deviation

### Differences in physical activity levels by gender and school grade

Statistical analyses of differences in accelerometry-related parameters revealed main effects of gender (except for sedentary behavior and light physical activity on weekends) and grade (except for moderate physical activity on weekends), as well as significant interactions between gender and grade (except for weekday and weekend step counts and weekend moderate physical activity) (Table 2). On weekdays, boys engaged in less sedentary behavior (F = 21.8, df = 1686, *p* < 0.01), more light (F = 14.8, df = 1686, *p* < 0.01), moderate (F = 10.1, df = 1686, *p* < 0.01), and vigorous (F = 7.7, df = 1682, *p* < 0.05) physical activity, and performed more total steps (F = 70.8, df = 1686, *p* < 0.01) than girls. On the weekends, boys also engaged in more moderate (F = 7.6, df = 1426, *p* < 0.05) and vigorous (F = 6.4, df = 1405, *p* < 0.05) physical activity, and took more steps (F = 22.6, df = 1427, *p* < 0.01) than girls. Higher-grade participants exhibited more sedentary behavior on weekdays (F = 232.8, df = 3686, *p* < 0.01) and weekends (F = 29.8, df = 3427, *p* < 0.01) than lower-grade participants. Higher-grade children were less active on weekdays, engaging in less light (F = 216.9, df = 3686, *p* < 0.01), moderate (F = 144.5, df = 3686, *p* < 0.01), and vigorous (F = 32.0, df = 3682, *p* < 0.01) physical activity than lower-grade participants. Higher-grade participants also took few steps on weekdays (F = 67.9, df = 3686, *p* < 0.01). Similarly, higher-grade students were less active on weekends, performing less light (F = 35.4, df = 3427, *p* < 0.01) and vigorous (F = 8.7, df = 3405, *p* < 0.01) physical activity, and taking fewer total steps (F = 4.4, df = 3427, *p* < 0.01) than lower-grade participants. Only moderate physical activity on weekends did not differ between higher- and lower-grade participants.

**Table 2:** Gender-specific physical activity and sedentary behavior levels by school grade

				Sedentary behavior (minutes/day)		Light physical activiy (minutes/day)		Moderate physical activity (minutes/day)		Vigorous physical activity (minutes/day)		Steps (steps/day)	
				Boys	Girls	Boys	Girls	Boys	Girls	Boys	Girls	Boys	Girls
Weekdays													
	Preschool	means		326.5	339.6	413.0	402.4	37.2	36.8	41.0	38.8	16552.9	14415.9
		95 % CI	lower	309.8	323.3	399.1	389.0	33.7	33.4	36.0	34.0	15479.0	13370.9
			upper	343.3	356.0	426.8	415.9	40.7	40.2	46.0	43.7	17626.8	15460.8
	Lower grades of elementary school	means		372.9	397.5	347.2	338.0	52.7	44.6	44.9	37.6	17757.4	13737.0
		95 % CI	lower	353.0	381.0	330.9	324.4	48.6	41.1	39.0	32.7	16489.0	12681.9
			upper	392.7	414.0	363.6	351.6	56.9	48.0	50.8	42.5	19025.8	14792.1
	Higher grades of elementary school	means		411.5	429.5	324.9	310.8	53.2	48.1	28.1	29.4	15540.2	13515.8
		95 % CI	lower	397.0	414.9	312.9	298.8	50.2	45.1	23.8	25.0	14612.3	12586.2
			upper	426.0	444.0	336.9	322.8	56.2	51.1	32.4	33.7	16468.0	14445.5
	Junior high school	means		474.8	519.9	286.2	251.8	27.5	26.9	29.2	19.6	12616.3	9199.2
		95 % CI	lower	465.7	510.9	278.7	244.4	25.6	25.0	26.5	16.9	12034.4	8622.5
			upper	483.9	528.9	293.7	259.2	29.4	28.8	32.0	22.3	13198.2	9775.8
Main effects	Gender	F		21.8		14.8		10.1		7.7		70.8	
		df		1686		1686		1686		1682		1686	
		*P* value		0.00		0.00		0.00		0.01		0.00	
		η2		0.03		0.02		0.01		0.01		0.09	
	Grade	F		232.8		216.9		144.5		32.0		67.9	
		df		3686		3686		3686		3682		3686	
		*P* value		0.00		0.00		0.00		0.00		0.00	
		η2		0.51		0.49		0.39		0.13		0.23	
Interaction	Gender × Grade	F		2.9		2.9		2.7		3.4		2.0	
		df		3686		3686		3686		3682		3686	
		*P* value		0.04		0.03		0.04		0.02		0.12	
		η2		0.01		0.01		0.01		0.01		0.01	
Weekends													
	Preschool	means		359.0	385.5	396.9	377.1	29.1	29.1	29.0	22.7	12762.5	11151.4
		95 % CI	lower	330.1	356.5	372.9	353.0	23.5	23.5	23.2	16.7	11226.3	9609.8
			upper	387.9	414.5	420.9	401.2	34.7	34.7	34.9	28.6	14298.7	12693.0
	Lower grades of elementary school	means		413.3	412.2	325.6	351.8	34.6	28.5	40.5	22.0	14014.2	9995.5
		95 % CI	lower	376.5	383.8	295.1	328.2	27.5	23.0	33.1	16.2	12061.0	8485.2
			upper	450.0	440.6	356.1	375.4	41.7	34.0	47.9	27.8	15967.4	11505.8
	Higher grades of elementary school	means		459.6	451.3	303.2	316.0	33.9	26.8	17.3	22.1	9924.6	9238.4
		95 % CI	lower	427.3	422.6	276.4	292.1	27.7	21.2	10.8	16.1	8204.3	7711.4
			upper	492.0	480.0	330.1	339.8	40.2	32.4	23.9	28.1	11644.8	10765.4
	Junior high school	means		461.1	520.7	301.7	252.4	34.6	26.0	18.0	17.0	12393.2	8359.6
		95 % CI	lower	438.0	502.5	282.6	237.3	30.1	22.5	13.2	13.1	11165.2	7395.3
			upper	484.2	538.8	320.9	267.4	39.0	29.5	22.9	20.9	13621.2	9324.0
Main effects	Gender	F		3.5		0.8		7.6		6.4		22.6	
		df		1427		1427		1426		1405		1427	
		*P* value		0.06		0.37		0.01		0.01		0.00	
		η2		0.01		0.00		0.02		0.02		0.05	
	Grade	F		29.8		35.4		0.2		8.7		4.4	
		df		3427		3427		3426		3405		3427	
		*P* value		0.00		0.00		0.88		0.00		0.00	
		η2		0.18		0.20		0.00		0.06		0.03	
Interaction	Gender × Grade	F		2.9		4.8		1.0		4.7		2.6	
		df		3427		3427		3426		3405		3427	
		*P* value		0.03		0.00		0.38		0.00		0.05	
		η2		0.02		0.03		0.01		0.03		0.02	

Adjusted for weight status and wear time

### Attainment of Physical activity guidelines by gender and grade level

Table 3 shows the odds ratios (ORs) of physical activity guideline attainment. Boys were more likely to meeting the recommendations than girls (OR: 2.07, 95 % confidence interval [CI]: 1.45–2.96). Preschoolers (OR: 6.66, 95 % CI: 4.01–11.06), lower-grade elementary school students (OR: 17.11, 95 % CI: 8.80–33.27), and higher-grade elementary school students (OR: 7.49, 95 % CI: 4.71–11.92) were significantly more likely to meet the guidelines than junior high school students.

**Table 3:** Odds ratios for meeting physical activity guidelines adjusted for weight status and accelerometer wear time

		% meeting guideline	OR	95 % CI	
Gender	Boys	59.0	2.07	1.45	2.96
	Girls	46.5	1.00		
Grade level	Preschool	72.4	6.66	4.01	11.06
	Lower grades of elementary school	86.4	17.11	8.80	33.27
	Higher grades of elementary school	75.0	7.49	4.71	11.92
	Junior high school	30.0	1.00		

*OR* odds ratio, *CI* confidence interval adjusted for weight status and wear time

## Discussion

The present study examined differences in objectively measured activity levels and attainment of physical activity guidelines in Japanese children and adolescents according to gender and school grade. Boys and younger students were more active than girls and older students. Moreover, approximately half of the preschool and elementary school boys and girls attained the recommended physical activity levels, while 70 % of junior high school students did not. These findings indicate that strategies to promote greater physical activity among Japanese children and adolescents should include special emphasis on physical activity for girls and on maintaining activity throughout adolescence.

The present results indicate that students engaged in less moderate and vigorous physical activity on weekends than weekdays, despite the time spent on mandatory sedentary weekday activities such as sitting in class. Similar trends were reported among young children and youth in Hungary [22], Germany [23], Portugal [24], China [25], France [26], Spain [27, 28], and Canada [29]. Thus, to promote moderate to vigorous physical activity, it may be effective to consider not only school-based approaches but also home and neighborhood-based approaches. Children have more time for physical activity on weekends at home or in a neighborhood setting than at school because there are time restrictions such as class on weekdays. Therefore, an approach that considers neighborhood environment, social support from family, and the community may increase physical activity among Japanese school children [30].

On both weekdays and weekends, boys were more likely to spend time on moderate and vigorous physical activities and less likely to spend time on sedentary behaviors than girls. The same was true of younger students compared to older students. Previous studies have shown that boys are more active than girls worldwide [9, 23, 24, 26–29, 31–40]. In Japan, the gender difference among elementary school children is especially pronounced. Therefore, promoting physical activity for elementary school girls is urgently needed. Moreover, the present results show children’s physical activity declines from elementary school to junior high school even though physical activity level was higher in elementary school than preschool. Previous studies from England [37], nine European countries [41, 42], France [26], U.S. [9, 34, 43], Canada [33], Spain [27], Denmark [7], and Mexico [44] have also shown that physical activity levels decline with age. Since physically active children are more likely to become physically active adults [45], it is important to identify ways to prevent the decline and to promote activity during the childhood years.

Time spent on sedentary behavior increased with school grade, and girls were more sedentary than boys on both weekdays and weekends. Similar results have been reported in previous studies [23, 26, 27, 43]. Moreover, children who spent less time on sedentary behavior spent more time on light physical activity, and vice versa, on both weekdays and weekends. These reciprocal relationships were found in previous studies as well [21, 33, 43], suggesting that a strategy focused on reducing sedentary behavior would concomitantly promote light physical activity. Therefore, in addition to strategies for promoting moderate to vigorous physical activity, strategies for reducing sedentary behavior and promoting light physical activity may benefit sedentary or slightly active children. On weekdays, sedentary behavior increases with grade because children in higher grades spend more time at school. On weekends, children are not restricted by class time. Therefore, a targeted plan to reduce sedentary behavior on weekends or outside of class time on weekdays, such as during recess or after school, may be effective.

Boys and younger students also took more steps per day than girls and older students on both weekdays and weekends. Boys were more likely to take the recommended number of steps [6] on both weekdays and weekends, whereas girls were likely to be 1000 steps per day under the recommended number [6] with the exception of preschool girls on weekends. Children take fewer steps per day on weekends than weekdays in other countries as well [46, 47]. A positive association was found between children’s physical activity on weekends and family encouragement or family social support [48]. Moreover, the Canadian Physical Activity Levels Among Youth (CANPLAY) study found that every 1000-step increase in a parent’s step count per day was associated with 200–450 extra steps per day in the child’s count [49]. Therefore, to increase steps on weekends, a family-based education program or intervention could be effective.

The present study indicated that boys were more than twice as likely to meet the WHO guidelines than girls, in agreement with previous studies on gender differences in activity goal attainment [23, 27, 35, 40–43, 50, 51]. For instance, fourth- through sixth-grade boys in the U.S. [43] were 5.85 times more likely than girls to attain recommended levels of physical activity. Also consistent with the present results, goal attainment also fell with age; fourth-grade children in the U.S. were 1.53 times more likely than fifth- and sixth-grade children to attain the guidelines. There are, however, substantial differences in goal attainment levels across studies. In the present study, less than 50 % of the children met the recommendation for at least 60 min of moderate to vigorous physical activity per day. However, a study in Europe found that more than 90 % of children attained the guidelines [43], while other studies in England [35], China [25], and European countries [50] found guideline attainment under 10 %. In the present study, 75.0 % of elementary school students in the higher grades met the guidelines, higher than the 24.3 % reported in the aforementioned U.S. study [43]. Guinhouya et al. reviewed several studies of European children and suggested that reported differences in guideline attainment could be attributed to different accelerometer cutoff points [52]. Up to 100 % of youth may be considered sufficiently active when using approximately >1000–1500 cpm, the most widely used cut-off point, up to 87 % when >2000 cpm is used, but no more than 3–5 % when a cut-off point of >3000 cpm is used. In the present study, the accelerometer was only capable of measurements for 2-min epochs, and it estimated rather than counting the minutes for each activity level and steps. Therefore, these percentages should be compared and interpreted carefully.

In 2012, the Ministry of Education, Culture, Sports, Science and Technology of Japan recommended that preschoolers participate in 60 min or more of physical activity daily [21]. Physical activity guidelines for older children have not yet been established in Japan. The present study indicates that the percentage of Japanese children attaining the guidelines is low. A better understanding of the sociodemographic, psychological, social, and environmental variables that affect guideline attainment is needed in order to develop effective strategies to encourage physical activity.

Some limitations of the current study should be considered. First, the accelerometer was capable of activity measurement only in 2-min epochs, while a shorter epoch is strongly recommended for children because their physical activity is often intermittent [53]. Therefore, the amount of sedentary time and vigorous physical activity may be underestimated while light and moderate physical activity may be overestimated. Second, the participants were living in a relatively narrow area of Japan, which could limit generalizability. Despite these limitations, the present study is meaningful as it is the first to indicate gender and age (school grade) differences in physical activity, sedentary behavior, and guideline attainment among children and adolescents in Japan. The present data can be used to inform interventions for promoting physical activity in Japanese children and adolescents.

## Conclusions

Japanese children and adolescents were engaged in sedentary behavior for more than half of the time they wore the accelerometer. Boys and lower-grade students were less sedentary and engaged in more physical activity than girls and higher-grade students. These findings highlight the need for effective and sustainable strategies to promote physical activity in Japanese school-age children.

### Availability of supporting data

There are no supporting data presented and linked with this study. Supporting data is available from the authors on request.
